# The leading global health challenges in the artificial intelligence era

**DOI:** 10.3389/fpubh.2023.1328918

**Published:** 2023-11-27

**Authors:** Amal Mousa Zaidan

**Affiliations:** ^1^King Saud bin Abdulaziz University for Health Sciences, Riyadh, Saudi Arabia; ^2^King Abdullah International Medical Research Center (KAIMRC), Riyadh, Saudi Arabia; ^3^Ministry of National Guard Health Affairs, Riyadh, Saudi Arabia

**Keywords:** global health, artificial intelligence, public health, global health challenges, machine learning, health care, mortality

## Abstract

Millions of people’s health is at risk because of several factors and multiple overlapping crises, all of which hit the vulnerable the most. These challenges are dynamic and evolve in response to emerging health challenges and concerns, which need effective collaboration among countries working toward achieving Sustainable Development Goals (SDGs) and securing global health. Mental Health, the Impact of climate change, cardiovascular diseases (CVDs), diabetes, Infectious diseases, health system, and population aging are examples of challenges known to pose a vast burden worldwide. We are at a point known as the “digital revolution,” characterized by the expansion of artificial intelligence (AI) and a fusion of technology types. AI has emerged as a powerful tool for addressing various health challenges, and the last ten years have been influential due to the rapid expansion in the production and accessibility of health-related data. The computational models and algorithms can understand complicated health and medical data to perform various functions and deep-learning strategies. This narrative mini-review summarizes the most current AI applications to address the leading global health challenges. Harnessing its capabilities can ultimately mitigate the Impact of these challenges and revolutionize the field. It has the ability to strengthen global health through personalized health care and improved preparedness and response to future challenges. However, ethical and legal concerns about individual or community privacy and autonomy must be addressed for effective implementation.

## Introduction

Millions of people’s health and well-being are at risk of several factors and multiple overlapping crises, including but not limited to infectious disease outbreaks, rising malnutrition rates, and lack of sufficient medical access; all hit the vulnerable the most (1). As we are heading toward the end of 2023, a record 339 million people globally need urgent aid. Several critical issues need to be addressed urgently to improve health globally and build resilience against future threats (1); the recent COVID-19 has shown that each country’s security and prosperity depend on creating a healthier, safer, more resilient world.

**Figure 1:**
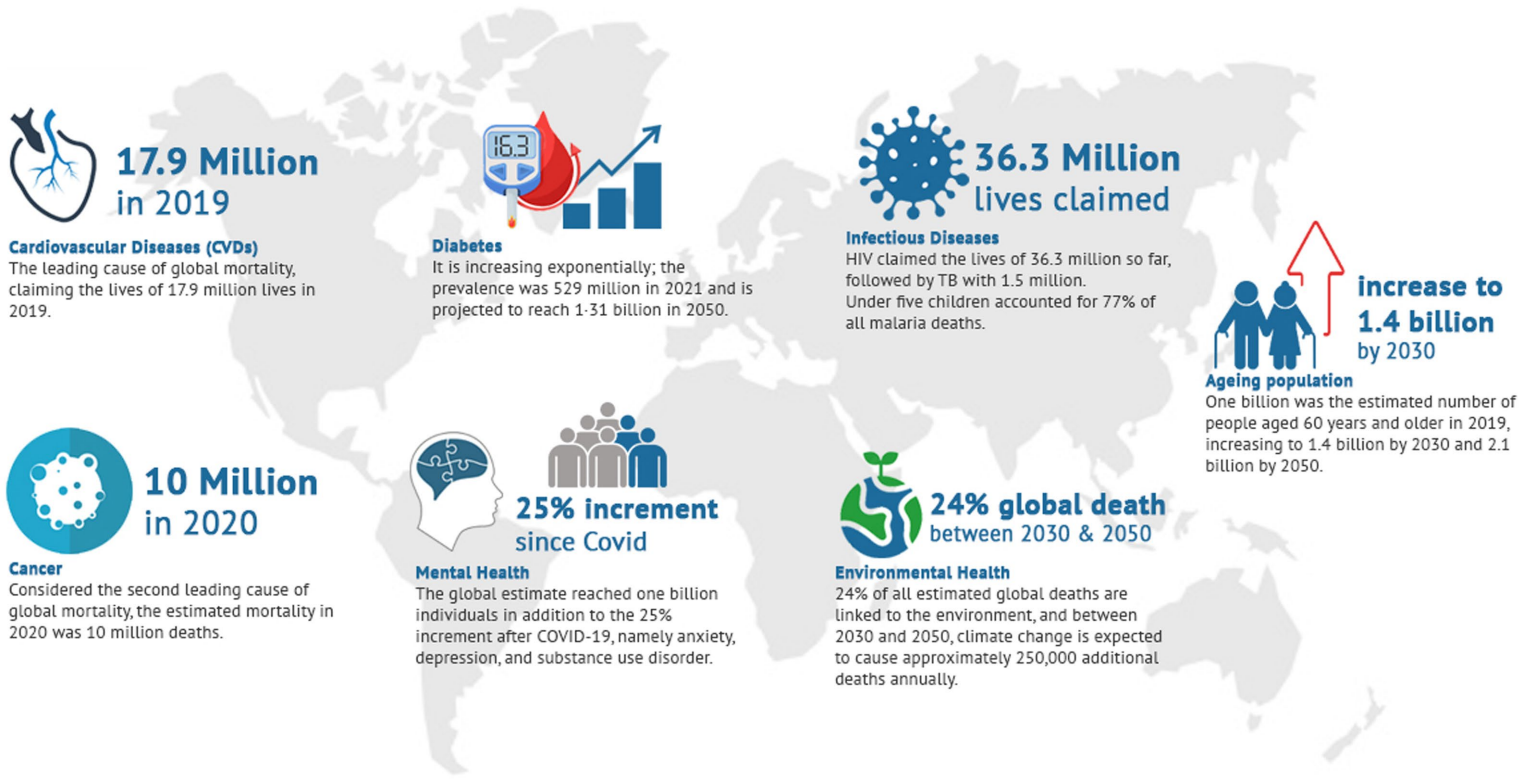
Examples of global health challenges.

Global public health priorities play a crucial role in addressing the most pressing health challenges faced by populations worldwide. They are dynamic and evolve in response to emerging health challenges and crises, which need effective collaboration among countries to secure global health. Mental Health, the Impact of climate change, cardiovascular diseases (CVDs), diabetes, Infectious diseases, health system, and population aging are examples of challenges that are examples of these challenges that are known to pose a vast burden worldwide (Figure 1) (2, 3).

We are at a point known as the “digital revolution,” characterized by the expansion of artificial intelligence (AI) and a fusion of technology types; the rapid and transformative changes brought about by these advances in digital technology led to increased connectivity and accessibility of vast amounts of information (4). AI has emerged as a powerful tool for addressing various challenges, and the last ten years have been influential in the digital health (5, 6). AI and its subfields or techniques, such as deep learning (DL), natural language processing (NLP), and machine learning (ML), have prospects to benefit healthcare, including public health, because of the rapid expansion in the production and accessibility of health-related data (7–9). The computational models and algorithms can understand complicated health and medical care data to perform various functions and deep-learning strategies. AI in health care improves disease surveillance, diagnosis, treatment selection, and clinical laboratory testing (10, 11). Harnessing its capabilities can ultimately mitigate the impact of global public health issues and revolutionize the field.

However, the burgeoning interest was accompanied by caution over using it, especially in health-related fields. Crucial ethics, privacy, and bias issues were raised to ensure AI’s responsible and equitable integration in the global public health landscape. Additionally, the human inability to see how ML systems make their decisions “black box,” brought uncertainty and threatened trust among the users regarding its application (12). This review aims to explore the current state of AI in those mentioned global public health challenges and provide insights about its current application in disease diagnosis, medicinal product development, and medical intervention. This step will provide more comprehension, and interpretability for most of AI’s leading evidence-based applications and contribute to a better understanding of AI in dealing with the increasing scale and complexity of challenges to global health.

## AI in non-communicable diseases

The global development over the last years was also associated with the change in disease burden, and NCDs have almost become the leading cause of mortality, resulting in 200 million premature deaths and expectation of another 150 million people deaths during the next ten years; most of them in low and middle-income countries (13).

NCDs are usually multifactorial diseases, and several factors are associated with their development, including genetic and environmental factors, which make them challenging to prevent and treat effectively. They are exacerbated by four key modifiable risk factors, namely tobacco use, harmful use of alcohol, physical inactivity, and unhealthy diets (14). It was estimated that behavioral factors and genetic factors are the main contributors to preterm death in the USA with (40%), and (30%) respectively (15), and the role of public health awareness and intervention have a vast impact (16). AI can offer the potential to analyze large and complex datasets obtained from lifestyle, clinical, and biological data in a way that exceeds the human ability to make sense of it. ML, NLP, Robotic, virtual agents (chatbots), and speech analysis are just a few examples of the available AI applications that are used to improve public health (17). For example, *Florence*, which is the WHO’s first virtual health worker, is designed to help the world’s 1.3 billion tobacco users quit smoking (18); *STop obesity Platform* that can offer personalized support to people with obesity (19), and *chatbots* to personalized fitness strategies.

If we discuss kidney disease progression as a case illustration, AI has its application in pre/post-diagnosis, which can ultimately lead to improved outcomes in a timely and accurate manner (20). During the analysis phase of patient data, AI can identify the early signs of the diseases from the lab results, medical history, and images (21, 22), it can also help in the diagnosis of kidney disease from a kidney biopsy through deep learning-based approaches (23), and to improve outcome and early detection of other comorbidity in renal patients (24). The flowing part discusses AI’s applications in the major of NCDs.

## AI in cardiovascular diseases

Cardiovascular diseases (CVDs) are the leading cause of global mortality, taking an estimated 17.9 million lives in 2019. Strategies to tackle this considerable burden are directed toward reducing risk factors, enhancing the health system, and monitoring disease patterns and trends to inform national and global actions. The application of AI can identify, process, integrate, and analyze massive amounts of data, including but not limited to medical records, ultrasounds, medications, and experimental results. The gold standard in diagnosing most CVDs is an echocardiogram (ECG) and cardiac magnetic resonance (CMR). However, the clinician’s interpretation of ECG depends on their experience. Hence, ECG information might be missed due to clinicians’ difficulty analyzing them (25, 26).

Additionally, most ECGs are done on symptomatic patients, even though many CVDs, such as valvular heart diseases, involve long asymptomatic periods (27). However, the AI algorithm interpretation of ECG can diagnose heart failure, atrial fibrillation, hypertrophic cardiomyopathy, pulmonary hypertension, aortic valve stenosis, and anemia (28–36). An approach that reduced time and the physician’s cognitive burden by offering pre-diagnosis, correcting clinician errors and preventing the occurrence of misdiagnosis. Moreover, AI enhances the prompt efficiency of medical tools such as computed tomography (CT), echocardiography, magnetic resonance imaging (MRI), and Coronary computed tomography angiography (CCTA) (37–39). In addition to its application in CVD prediction and predictive modeling, such as mortality prediction, vascular aging, and predicting major adverse cardiovascular events in asymptomatic subjects (40–43).

## AI in cancer

Cancer is a large group of diseases and is considered the second leading cause of global mortality; the estimated mortality in 2020 was 10 million deaths. Despite the several accomplishments that have been made in the field of cancer diagnosis, prognosis, and treatment, individualized and data-driven care remains challenging. The challenge lies in the specific characteristics of distinct molecular, genetic, and tumor-based features (44). However, using machine learning and AI positively supports cancer prevention and management, as it is reshaping the existing picture, and is developing rapidly (45–47). AI has become pivotal as it can provide patients with forecasting and prediction and improved risk stratification according to specific criteria, such as in the cases of some breast, colon, ovarian, lung, and skin cancers (48–54). Further, it can detect hidden patterns from several sources such as molecular profiling, pathology, and medical imaging, and integration of-omics data to provide a more comprehensive understanding of cancer and improve the precision oncology (55). During a surgical procedure, AI can provide real-time detection and diagnosis of some cancers; through its ability to differentiate between cancerous and normal tissues (56, 57). Recently, mounting evidence indicated the potential role of microRNAs (miRNA) in cancer diagnosis and prognosis. MiRNAs, a small, single-stranded, non-coding ribonucleic acids (RNAs), are essential for all biological functions including cancer development. ML provides an opportunity to explore miRNA’s ability to serve as a reliable biomarker targeting drugs and improve cancer clinical classification (58, 59).

A recent achievement is the genetics-based classification and treatment response of Cancer of unknown primary (CUP); this type of cancer usually leads to poor outcomes because primary cancer is unknown (60). The study used ML to classify the cancer based on its genetic profile. This model identifies the likely prior site and predicts the best treatment option (60). Another example is pancreatic cancer, one of the most challenging cancers to diagnose as it is often asymptomatic until it metastasises, causing poor and ineffective treatment. AI modeling enables the detection of individuals at high risk of developing pancreatic cancer; the detection was up to 3 years earlier than currently by using medical records (61).

## AI in diabetes

A global crisis that is increasing exponentially and is considered a significant cause of blindness, kidney failure, heart attacks, stroke, and lower limb amputation. It was estimated that the prevalence was 529 million in 2021 and is projected to reach 1·31 billion in 2050 (62), a burden that has a global agreement to halt before. According to several recommendations, the starting point is prevention by screening, especially for obese or overweight adults (63, 64). However, a significant number of cases were missed with these approaches. The current clinical application of AI in diabetes diagnosis and management is categorized into four domains: (1) automatic retinal screening, (2) clinical diagnosis support, (3) patient self-management tools, and (4) risk stratification (65). AI for automatic retinal screening enables early diagnosis with high specificity and sensitivity (66). Several studies have evaluated the prediction of new-onset diabetes mellitus by AI and ML models, and it was recommended to include data as an omics database (e.g., genomics) (67). Recently, a new model based on AI was developed to detect diabetes warning signs, even in patients who did not meet the guidelines for diabetes elevated risk. This model can enhance type 2 diabetes (T2D) detection; it uses the patient’s X-ray image collected during routine medical care and their medical records to detect T2D (68).

In diabetes management, AI devices can help patients monitor their glucose levels in real-time and predict spikes or drops in healthcare. AI-based medical devices, such as the Guardian Connect System by Medtronic and the DreaMed Diabetes system (DreaMed Diabetes Ltd), have been approved to help control diabetes (69, 70). Another mobile application (GoCARB) is used to estimate the carbohydrate content in meals, which can help enhance the patients’ skills in managing diabetic disease through diet management (71, 72). The future application of AI will introduce a paradigm shift in Diabetes care from conventional management to more personalized and data-driven precision care.

## AI in population aging

As a result of significant increases in life expectancy, the global population of people aged 60 years and older is increasing (73). One billion was the estimated number in 2019, rising to 1.4 billion by 2030, accounting for 16.7% of the global population, and projected to reach 2.1 billion by 2050; around 80% will live in low and middle-income countries (74, 75). The increase in age is associated with common health conditions, leading to several complex health states due to multiple underlying factors, such as disability. However, there is no linearity or consistency in developing these changes, and individual characteristics have a vast impact. They will continuously demand primary and long-term care, a more trained workforce, and physical and social environments for social support. Around 92% of global older adults have at least one chronic disease, and more than 81% of those aged ≥85 years suffer from two chronic diseases or more (76–78). Additionally, disability and its consequences also have a huge burden on the aging population. Globally, 1.3 billion people (16% of the world’s population) suffer from a physical or cognitive disability; although these estimates cover all age groups, starting from 18 years old (79). However, AI can provide intelligent solutions for longer lives, satisfy the growing unmet healthcare needs, and overcome the limited number of insufficient healthcare resources. Currently, AI technologies for aging population are used in the robotic intervention (80, 81), applications on smartphones or computers (82, 83), social interaction and support, such as improved mental well-being and quality of life (84–86); rehabilitation therapy, such as its application in the recovery of upper and lower extremity functions, gait robotic rehabilitation, or improve sleep quality and daily living activities (87–90), and wearables, voice-activated (91–94). It can create more advanced algorithms to provide more precise holistic interventions tailored to address the elders’ multiple needs in a safer and more friendly manner (78, 95, 96). Ambient-assisted working and ambient-assisted living are examples of smart systems that can adapt themselves to older adult needs by exploiting ambient intelligence solutions. These systems focus on using technology to support and enhance the quality of life of the older adult population, either in work, or indoor and outdoor environments (97, 98). Recently, a group of researchers have helped develop drugs that might potentially delay the effects of aging by eliminating senescent cells (96, 99).

## AI in mental health

The global estimate of mental health disorders is one billion individuals (100). Since the beginning of the COVID-19 pandemic, the rates of anxiety, depression, and substance use disorder have increased (101, 102). This situation is worse in low-and middle-income countries, where the estimated number of people with limited access and no treatment is around 75% of people with mental, neurological and substance use disorders (100, 102).

Despite the significant advantages of using AI in healthcare, mental health has been slower to adopt AI since the primary factor contributing to successful psychiatric diagnosis and treatment is the interaction with the patients’ (103–105). However, AI applications have great potential in diagnosing different kinds of mental illness. This is a great advantage given the available heterogeneity in the pathophysiology of mental illness. AI can access and analyze relevant information about a patient’s unique bio-psycho-social characteristics and identify pertinent data patterns that might help provide more objective, improved definitions of these illnesses (106). Further, AI can be used in biomarkers identification, develop better diagnoses and formulate risk models to predict individual risk (105, 107). Moreover, it can be used for some cases, such as depression or autism, where face-to-face interaction might be challenging. In autism, for example, AI could be a more useful tool than a psychotherapy session with a human doctor; it can provide tailored, personalized interventions or bridge the communication gap they may experience (105). Nevertheless, the variation of AI applications is persistent in dealing with sensitive issues like mental health.

## AI in infectious diseases

As the world becomes increasingly globalized, health and illnesses have no borders. Concerns about One Health have gained prominence recently, which is justified as the world emerges from the most significant global emergency and the increasing number of infectious pathogens that spread from humans, animals, or the environment. COVID-19 has highlighted the high spreading rate with which infections can devastate the world’s health and economy. Results in more investment and investigation into the occurrence, prevalence, prevention, control, and treatment of infectious diseases to strengthen the epidemic response and mobilize quickly for public health priority.

Globally, the leading communicable diseases associated with high mortalities are HIV/AIDS, tuberculosis (TB), malaria, viral hepatitis, sexually transmitted infections, and neglected tropical diseases (NTDs). HIV continues to be a major global issue, claiming the lives of 36.3 million so far, and TB-associated mortalities reached 1.5 million annually, making it the world’s second top infectious killer after COVID-19 (108). Tackling AI applications in HIV will yield several examples in HIV prevention, testing, and treatment to achieve sustained viral suppression (109–111). It was used also for rapid detection and response through monitoring clusters of vulnerable groups to reduce HIV transmission (111). Another example is Syphilis, which is a sexually transmitted disease (STD). To eliminate congenital syphilis (CS), the WHO launched an initiative in Latin America and the Caribbean (112). However, as the syphilis epidemic increased in Brazil, the government of Brazil developed a national project, the “Syphilis No!” Project (SNP), for implementing and integrating a syphilis response into healthcare networks, (113, 114). This project encompasses four dimensions: (a) management and governance, (b) surveillance, (c) comprehensive care, and (d) strengthening of the educommunication (113, 115). The application of AI such as data mining and NLP in these strategies augments the country’s capabilities in combating syphilis (113).

It is essential to analyze global infectious disease cases regularly. However, some countries’ investment in contagious disease identification was typically based on the identification of presenting symptoms and the likelihood of exposure due to the high cost and feasibility of the primary approach of detection (116, 117). However, using big data, AI and ML algorithms can contribute to global infection control and help with the spatial and temporal prediction of the evolution and spread of infectious diseases (118). Their advanced capabilities can analyze several factors: population demographics, environmental conditions, and individual behaviors, all of which can be used simultaneously (119). Such as case prediction according to historical data (120), predicting the likelihood of an individual contracting an infectious disease according to personal and behavioral characteristics, using pathogen genetic makeup to identify the most likely sources of an outbreak, identifying or anticipating an epidemic by analyzing massive data; it can be used for early warning systems, hot spot detection, forecasting, and improving the recourses allocation at a country and a global level (68–72). After the exposure or presence of a potential outbreak, AI can advance in diagnostic approaches and differentiate various pathogens by using the pathogen genetic makeup, such as its ability to distinguish between COVID-19 and other circulating respiratory viruses with COVID-like symptoms (121, 122).

Another example is the possible application to the rising incidence of antimicrobial resistance (AMR), which has become a significant challenge. For this purpose, a group of researchers were able to develop a mobile application to classify bacterial susceptibility to various antibiotics, especially in resource-limited settings (123). Further, reducing transmission is essential to control global widespread infections such as those that occur in pandemics. The application of AI for screening technologies targeting infections and integrating them into data visualization has been introduced broadly, especially during the COVID-19 pandemic (117). This improved the surveillance and generated meaningful insights from multidimensional data, which can be widely used for public health practice.

In addition to surveillance, early detection, and diagnosis, AI is used to develop anti-infective therapies, although it became challenging with the spread of drug resistance (124). ML models can help explore the pathway of pathogen’s interaction with host cells and immune responses, facilitating antigen determination, vaccine design, and treatment strategies (124–127). Finally, the WHO global report on infection prevention and control estimated that implementing infection prevention and control (IPC) can reduce healthcare-associated infections (HAIs) by 70% (128). Using AI can improve current and past processes to speed infection prevention and control response, such as identifying the correlations associated with medically relevant conditions, identifying potential risk factors, and surveillance of emerging infectious diseases (129–131), improving hand hygiene compliance (132), and in-hospital analysis of transmission, and outbreak events identification and investigation (133).

## AI in environmental health

The impact of environmental health on human lives and health are interconnected in various ways. The Global Health Observatory estimated that 24% of all estimated global deaths are linked to the environment. Between 2030 and 2050, climate change is expected to cause approximately 250,000 additional deaths per year, mainly from undernutrition, malaria, diarrhea and heat stress (134). Because of the adynamic of the environment, AI applications in this field are immense; its deployment will provide a better capacity to deal with the growing climate exigency and related challenges. In exposure assessment, AI can use satellite observations, meteorological variables, land use, and traffic data to predict the spatiotemporal patterns and concentrations of pollutants (135–138). AI were used in monitoring, such as its application during COVID-19, for airport security checks and patient tracking (139), or to improve the prediction of harmful algal blooms (140).

Additionally, it can predict diseases based on environmental factors, such as its application to predict the spread of Zika virus and Dengue fever (141, 142). In waste management, AI reduces fuel consumption and emissions, increases recycling rates, and reduces landfill waste (143). GeoAI is one of the emerging AI tools that can handle complex spatial and temporal data to adjust algorithms and workflows according to the specific characteristics of spatial processes (136, 144). It can develop various environmental exposure models across different geographical regions in prospective and retrospective approaches (136). In 2022, the United Nations launched The World Environment Situation Room, a new digital platform that can provide real-time analysis, track air quality, measure environmental footprint, and monitor (145).

## AI in health systems

Through good stewardship, resource development, funding and services, health systems support initiatives to prevent, promote, and provide for more health and well-being (146). They are complex and are in a constant state of flux; according to the World Health Organization (WHO), “A well-functioning health system working in harmony is built on having trained and motivated health workers, a well-maintained infrastructure, and a reliable supply of medicines and technologies backed by adequate funding, strong health plans and evidence-based policies” (147). At the global level, it should be able to control and address global health challenges and severe events (147). However, several myriads of difficulties impede their ability to provide these services. This includes but is not limited to the sudden onset or the slowly growing crises, such as the COVID-19 pandemic, the natural disasters the world is encountering, or the slow time impact of climate change (148–150), the rising number of older people, and the associated complex chronic medical illness. To overcome these difficulties and achieve effective and lasting change, four factors were proposed: (a) the acorn-to-oak tree principle (small initiative), (b) the data-to-information-to-intelligence principle (information technology (IT) and data), (c) the many-hands principle (stakeholders); and (d) the patients-the-preeminent-player principle (individuals) (151). These factors were established across 60 health systems; the role of data and technology cannot be missed (5, 151). AI applications are steadily entering novel domains previously governed solely by human experts. They can improve health financing, make public health more effective, and reach underserved populations by making health care more efficient and effective through more personal health services (152).

Further, the Primary health care system (PHC) is vital to addressing health issues effectively; they are considered the front door of the health care system. Using AI will enhance the holistic approach of PHC in outcome prediction, data mining, and personalized treatment (153–155). The current tools in PHC have several applications, including the risk prediction (156–158), workforce assessment (159), record data extraction (160, 161), control of healthcare-associated infections (162), and performing medical tasks remotely that contribute to public health domain (154, 163, 164).

## Conclusion

AI integration and application to global health challenges have immense potential to overcome them efficiently and effectively. Disease prevention, detection, and response can quickly mobilize and yield medicinal products. As mentioned earlier, around 40% of preterm death was associated with behavioral factors. With AI advancement, data analysis and segmentation can be done for several characteristics such as behavior, opinion, and attitude. Using these data, the ML can analyze the online health information and provide personalized massaging to influence individuals’ health behaviors with high quality and clarity, amplifying their influence and effectiveness (165–167). This health communication can also inform AI technology in developing effective communication systems with patients and their healthcare providers. The health communication theories and models can highlight the available barriers to behavioral change and the available limitations of technology-driven health interventions. Which can help improve the efficacy of AI-supported systems or intervention designs (167–169).

AI in health care is expected to grow from nearly US $15 billion to $103 billion between 2023 and 2028 (170). However, incongruent with AI’s benefits, exacerbation of inequities was accompanied, and ethical and legal concerns about individual or community privacy and autonomy were raised (118, 171). The EU AI Act is nearing implementation, and it will be the first comprehensive regulation that addresses the risks of artificial intelligence; European Parliament proposed it to ensure better conditions for developing and using this innovative technology (172). Further, to avoid the risk of hindering AI applications in healthcare due to lack of sufficient transparency “black box,” researchers were urged to provide more research and explanation for AI; explainable AI (xAI), as an approach to more understandable and human-interpretable AI-based applications (173, 174).

However, AI stands as a cornerstone of the upcoming digital revolution. Despite the moral dilemmas in AI application in health care, it is likely to meager, co-exist or replace current systems and assets as a potent amplifier of human potential. It has the ability to strengthen global health through personalized health care and improved preparedness and response to future challenges.

## Author contributions

AZ: Conceptualization, Writing – original draft.
